# Excellent local control and tolerance profile after stereotactic body radiotherapy of advanced hepatocellular carcinoma

**DOI:** 10.1186/s13014-017-0851-7

**Published:** 2017-07-12

**Authors:** Eleni Gkika, Michael Schultheiss, Dominik Bettinger, Lars Maruschke, Hannes Philipp Neeff, Michaela Schulenburg, Sonja Adebahr, Simon Kirste, Ursula Nestle, Robert Thimme, Anca-Ligia Grosu, Thomas Baptist Brunner

**Affiliations:** 1 Department of Radiation Oncology, University Medical Center, Freiburg, Germany; 2 Department of Gastroenterology, Hepatology, Endocrinology and Infectious Diseases, University Medical Center , Freiburg, Germany; 3 Department of Radiology, University Medical Center, Freiburg, Germany; 4 Department of General and Visceral Surgery, University Medical Center, Freiburg, Germany; 5 Department of Nuclear Medicine, University Medical Center, Freiburg, Germany; 6 German Cancer Consortium (DKTK), Partner site Freiburg, Freiburg, Germany; 7 Faculty of Medicine, University of Freiburg, Freiburg, Germany; 8 German cancer Research Center (DKFZ), Heidelberg, Germany; 9 Berta-Ottenstein-Programme, Faculty of Medicine, University of Freiburg, Freiburg, Germany

**Keywords:** Stereotactic body radiotherapy, SBRT, Hepatocellular carcinoma, HCC, SIP

## Abstract

**Background:**

To evaluate the efficacy and toxicity of stereotactic body radiotherapy (SBRT) in the treatment of advanced hepatocellular carcinoma (HCC).

**Material and Methods:**

Patients with large HCCs (median diameter 7 cm, IQR 5-10 cm) with a Child-Turcotte-Pugh (CTP) score A (60%) or B (40%) and Barcelona-Clinic Liver Cancer (BCLC) classification stage B or C were treated with 3 to 12 fractions to allow personalized treatment according to the size of the lesions and the proximity of the lesions to the organs at risk aiming to give high biologically equivalent doses assuming an α/β ratio of 10 Gy for HCC. Primary end points were in-field local control and toxicity assessment.

**Results:**

Forty seven patients with 64 lesions were treated with SBRT (median 45 Gy in 3–12 fractions) with a median follow up for patients alive of 19 months. The median biological effective dose was 76 Gy (IQR 62–86 Gy). Tumor vascular thrombosis was present in 28% and an underlying liver disease in 87% (hepatitis B or C in 21%, alcohol related in 51%, nonalcoholic steatohepatitis in 13% of the patients, primary biliary cirrhosis 2%). Eighty three percent received prior and in most cases multiple therapies. Local control at 1 year was 77%. The median overall survival from the start of SBRT was 9 months (95% CI 7.7–10.3). Gastrointestinal toxicities grade ≥ 2 were observed in 3 (6.4%) patients. An increase in CTP score without disease progression was observed in 5 patients, of whom one patient developed a radiation induced liver disease. One patient died due to liver failure 4 months after treatment.

**Conclusion:**

SBRT is an effective local ablative therapy which leads to high local control rates with moderate toxicity for selected patients with large tumors.

**Electronic supplementary material:**

The online version of this article (doi:10.1186/s13014-017-0851-7) contains supplementary material, which is available to authorized users.

## Background

Liver cancer is the seventh most common cancer worldwide, with more than 782,000 new cases diagnosed in 2012 (6% of the total) and the second most common cause of cancer-related deaths worldwide after lung cancer, with more than 745,000 deaths annually [1].

Surgery is the mainstay of HCC treatment with a 5 year overall survival of 50% [2], yet only 15% of the newly diagnosed HCC-patients are eligible for surgery [3], transplantation or radiofrequency ablation (RFA). These treatment options are limited due to impaired liver function including portal hypertension, advanced stage of HCC or other medical contraindications.

In intermediate stage HCC, according to the Barcelona Clinic Liver Cancer staging system (BCLC B) [4], with a multinodular affection transarterial chemoembolization (TACE) is the gold standard therapy to date. TACE was shown in randomized trials to improve survival compared with symptomatic therapy alone, in patients without macrovascular involvement. In advanced stage HCC (BCLC C), including HCC with portal invasion or metastasis and refractory disease following TACE, Sorafenib is the only systemic treatment option. [5].

Traditionally, the use of conventional external beam radiation therapy in HCC treatment was limited due to the low radiation tolerance of the liver especially in patients with underlying disease and a high Child-Turcotte-Pugh score. Yet in the recent years advances in treatment delivery and techniques allowed for enhanced delivery of ablative doses while sparing surrounding critical tissues using stereotactic body radiotherapy (SBRT). Radiation induced liver disease (RILD) after SBRT occurs in fewer than 5% of cases with careful patient selection [6].

Several prospective and retrospective trials showed promising results in patients treated with SBRT with high rates of local control and acceptable toxicity [7–11]. Thus SBRT could be considered as an alternative to ablation and/or embolization techniques or in cases were these therapies have failed or were contraindicated [12]. Nevertheless there is limited data concerning the safety of SBRT for large tumors or patients with impaired liver function.

In this study we evaluated the efficacy and toxicity of SBRT in large HCC tumors and impaired liver function unsuitable for other treatment options.

## Methods

### Patients and treatment characteristics

This retrospective analysis was approved by the institutional research ethics board. All consecutive patients with HCC, treated with SBRT between 2012 and 2015, who were unsuitable for surgery, RFA or TACE after multidisciplinary board (MDT) decision were enrolled in this analysis and treated according to the same institutional standard operating procedures (SOPs). The MDT panel typically indicated SBRT in patients with very large lesions that progressed or were not suitable for other local treatment modalities taking into account the relative position with respect to organs at risk for SBRT. For patients (BCLC stage B), with progressive disease (PD) after TACE, SBRT was offered as a local ablative option, as an alternative to systemic therapy with sorafenib, which has a high toxicity profile. Additionally patients with BCLC stage C with PD under sorafenib had no other treatment options.

Diagnosis was established either based on imaging techniques and/or by biopsy [2]. All patients had a good Eastern Cooperative Oncology Group (ECOG) performance status ≤2 with a life expectancy ≥6 months. Clinical examination, blood samples including liver scores and α-fetoprotein and the evaluation of the Barcelona-Clinic Liver Cancer (BCLC) classification, Child-Turcotte-Pugh (CTP) score were assessed before SBRT for all patients.

### SBRT techniques

All patients were immobilized in supine position with a customized vacuum cushion (BlueBAG BodyFIX, Innovative Technologies Völp, Innsbruck, Austria) and underwent 4 dimensional-CT (4D CT, Brilliance CT Big Bore, Philips Medical Systems, Cleveland, OH). For the 4D acquisition, breathing motion was monitored (Mayo Clinic Respiratory feedback system), including abdominal compression to minimize respiratory motion, using a phase based binning method with ten respiratory phases. The gross tumor volume (GTV) was defined as the arterial phase enhancing lesions with washout in the venous phase and/or delayed phase CT and MRI. Tumor vascular thrombosis (TVT) was also included into the GTV. The internal target volume (ITV) was created accounting for the extent and the position of the tumour at all motion phases in 3 dimensions using the 4D–CT image data. The PTV was a uniform 4 mm expansion of the ITV in all dimensions. Either fiducial markers were implanted or lipiodol depositis, surgical clips, transjugular intrahepatic portosystemic stent shunting (TIPPS) stents were used as fiducial markers for image guided radiotherapy (IGRT). In limited cases where the use of fiducial markers was contraindicated and the tumor was located in the right superior or inferior pole of the liver, the ITV of the borders of the liver was used for IGRT. Patients were treated with 3 to 12 fractions, dependent on the proximity to the organs at risk (OARs) (stomach, small intestine, colon and duodenum) delivered every other day. Three fraction regimens (typically 3 × 12.5–15 Gy) were preferred in patients with lesions away from critical structures, 12 fraction regimens (typically 12 × 4–5.5 Gy) were preferred in patients with contact to OARs, and 5 fraction regimens (typically 5 × 7–10 Gy) were intermediate in terms of closeness to OARs. For lesions where dose constraints as proposed by Timmerman et al. [13] could not be achieved, we utilized a simultaneous integrated protection (SIP) dose prescription, an intensity modulated radiotherapy (IMRT) technique described in detail elsewhere instead of reducing the dose to the entire PTV [14]. From 2007 to 2013 treatment was prescribed either to the 60 or 80% encompassing isodose and thereafter according to ICRU report 83 with a D_max_ of 110–120%. For analysis the prescribed doses were converted to biological effective doses (BED) and equieffective doses for 2 Gy fractions (EQD2), assuming that tumour and late reacting bowel tissue α/β ratios were 10 Gy and 3 Gy, respectively [15].

For all patients a daily on-line correction using cone beam computed tomography (CBCT) scans was applied and oral contrast was given to visualise stomach and/or duodenum in cases of close proximity.

### Toxicity and follow up

Patients were clinically examined at least weekly during treatment by radiation oncologists. During follow up, complete history, physical examination, blood tests and triphasic liver CTs or MRIs were acquired every three months. Toxicity was scored using the NCI Common Terminology Criteria for Adverse Events v4.03. Radiation induced liver disease (RILD) was defined according to Pan et al. [16] with the typical occurrence between one week to three months after treatment in the absence of PD. All toxicities reported within 3 months after treatment completion were considered as acute; thereafter, any toxicity was considered to be late.

### Statistical analysis

The primary end points were toxicity assessment and local control (LC) in the PTV (‘in-field’) at 1 year; the latter was defined as the absence of PD within the PTV as per Response Evaluation Criteria in Solid Tumors (RECIST) v1.1 in multiphasic CT or MRI. Lesions that developed or progressed outside the PTV in the liver or lymph nodes were scored as regional PD and those developed in other organs as distant PD. Survival and control times were calculated from the start of SBRT. Time to progression and survival were evaluated with the Kaplan-Meier method. Analyses were performed using SPSS (SPSS Inc., Chicago, IL) Statistical significance was set to *p* ≤ 0.05 and both sided.

## Results

### Patients and treatment characteristics

Between 2013 and 2016, a total of 47 patients with 64 lesions were included in the analysis with a median follow up of for patients alive of 19 months. Patient and Treatment characteristics are summarized in Table 1. Twenty-three patients (49%) had a BCLC stage B and 24 (51%) patients a stage C at the time of the analysis. Twenty-eight patients (60%) had a CTP score A (A5 = 16, A6 = 12) and 19 (40%) a CTP score B (B7 = 10, B8 = 6, B9 = 3). Nine (19%) patients were diagnosed with oligometastatic disease (lung = 4, bones = 4, adrenal *n* = 2) prior to SBRT, 38 (81%) patients had multifocal disease confined to the liver and five patients had regional lymph node metastases. Ten patients were treated with SBRT at two different HCC lesions simultaneously and seven patients were pre-treated with SBRT for another HCC-lesion in the past at median time between the two treatments of 5 months. Seventy eight percent received prior and in most cases multiple therapies. Pretreatment included resection (9 patients), radiofrequency ablation (5 patients), sorafenib (10 patients), transarterial chemoembolization (TACE, 34 patients), SBRT (7 patients) and selective internal radiation therapy (SIRT, 2 patients). For the latter a moderate fractionation of 12 × 4 Gy was used for the SBRT, mainly due to the lesions size. The first patient had a tumor of 21 cm in maximum diameter and the second patient two lesions of 11 and 9 cm, respectively. Both patients had a CTP score of A5. No additional constraints were taken into consideration for patients who underwent SIRT or Re-SBRT.

**Table 1:** Patient and treatment characteristics

Variable	All patients	CTP A	CTP B
	No. (%)	No. (%)	No. (%)
No. of patients	47 (100%)	28 (60%)	19 (40%)
Age(years)			
Median (range)	69 (29–84)	70 (45–84)	69 (29–83)
BCLC stage			
B	23 (49%)	14 (50%)	9 (47%)
C	24 (51%)	14 (50%)	10 (53%)
CTP score			
A	28 (60%)		
A5	16 (34%)	16	
A6	12 (26%)	12	
B	19 (40%)		
B7	10 (21%)		10
B8	6 (13%)		6
B9	3 (6 %)		3
Underlying liver disease	41 (87%)		
Hepatitis B	4 (9%)	3 (11%)	1 (5%)
Hepatitis C	6 (13%)	3 (11%)	3 (16%)
Alcohol related	24 (51%)	11 (39%)	12 (63%)
NASH^a^	6 (13%)	5 (18%)	2 (11%)
Primary biliary cirrhosis	1 (2%)	1 (7%)	0 (0%)
None	6 (13%)	5 (18%)	1 (5%)
Previous treatments^g^			
Surgery	9 (19%)	8 (29%)	4 (21%)
TACE^b^	34 (72%)	21 (75%)	13 (68%)
RFA^c^	5 (11%)	5 (18%)	1 (5%)
Sorafenib	10 (21%)	8 (29%)	2 (11%)
SIRT^d^	2 (4%)	2 (7%)	0 (0%)
SBRT^e^	7 (15%)	2 (7%)	3 (16%)
Tumor vascular thrombosis	13 (28%)	5 (18%)	8 (42%)
Extrahepatic disease	9 (19%)	6 (21%)	3 (16%)
Multiple lesions at baseline	39 (83%)	24 (86%)	15 (79%)
GTV volume^f^			
Median (IQR) cm^3^	77 (37–229)	69 (35–214)	108 (38–268)
Diameter of the lesions^f^			
Media(IQR) cm	7 (5–10)	7 (4–10)	8 (5–10)
Liver Volume			
Median (range) cm^3^	1654 (1384–2230)	1492 (1340–2277)	1819 (1554–1997)
Prescription dose (Gy)^f^			
Median (IQR)	45 (38–48)	45 (38–48)	45 (44–50)
Dmax			
Median (IQR)	53 (49–59)	54 (52–59)	51 (48–58)
D95%			
Median (IQR)	45 (38–48)	45 (37–48)	45 (44–50)
EQD2_10_ prescribed (Gy)^f^			
Median (IQR)	63 (51–75)	56 (50–71)	71 (56–85)
EQD2_10_ Dmax			
Median (IQR)	86 (62–104)	91 (62–104)	82 (61–99)
EQD2_10_ D95%			
Median (IQR)	60 (52–76)	56 (48–72)	71 (56–85)
BED_10_prescribed (Gy)^f^			
Median (IQR)	76 (62–86)	67 (61–85)	86 (67–102)
BED_10_ Dmax			
Median (IQR)	102 (75–125)	109 (75–125)	99 (73–115)
BED_10_ D95%			
Median (IQR)	72 (62–91)	67 (57–86)	86 (67–102)
Mean liver dose (Gy)			
Median (IQR)	17.9 (8.7–25)	19 (9.8–25)	16.5 (7.8–22)
EQD2_2, mean liver dose_			
Median (IQR)	16.5 (9.5–23)	17.2 (9–23.5)	15.6 (8.5–20.5)

^a^nonalcoholic fatty liver disease

^b^TACE: transarterial chemoembolization

^c^RFA: radiofrequency ablation

^d^SIRT: selective internal radiation therapy

^e^SBRT: stereotactic body radiotherapy

^f^per lesion

^g^some patients had more than one treatments previous to SBRT

*IQR* inter-quartile range

Median GTV was 77 cm^3^ (interquartile range IQR 37 to 229 ml) at a median liver volume of 1654 ml (IQR 1384 to 2230 ml; Table 1). The median diameter of the lesions was 7 cm (range 1.7–22, IQR 5–10) cm. Tumor vascular thrombosis (TVT) was present in 13 (28%) patients. Some treatments were planned with inhomogeneous dose distributions: planning target volume encompassing doses were most frequently 80% (5 lesions, 8%) and 60% (12 lesions, 19%) of the maximum dose. The median prescribed SBRT dose was 45 (IQR 38–48) Gy in 3 to 12 fractions [see Additional file 1] with a median maximum dose (Dmax) of 53 (IQR 49–59) Gy. One patient discontinued treatment due to esophageal varices bleeding (outside the PTV) after 21 Gy. The median prescribed biological effective dose was (BED_10_) of 76 (IQR 62–86 Gy) and an equivalent dose in 2 Gy fractions (EQD2_10_) of 63 Gy (IQR 51–75 Gy). For IGRT fiducial markers were implanted in 6 patients, lipiodol deposits of prior TACE were used in 34 patients, surgical clips in 1 patient and the TIPPS stents were used in 2 patients. In four cases where the tumor was located in the right superior or inferior pole of the liver, where the implantation of fiducial makers was not possible, the ITV of the borders of the liver was used for IGRT. Twenty-five patients were treated with SIP. Volumetric Arc IMRT was performed in 45 patients.

### Local control per lesion and patterns of failure

**Fig. 1:**
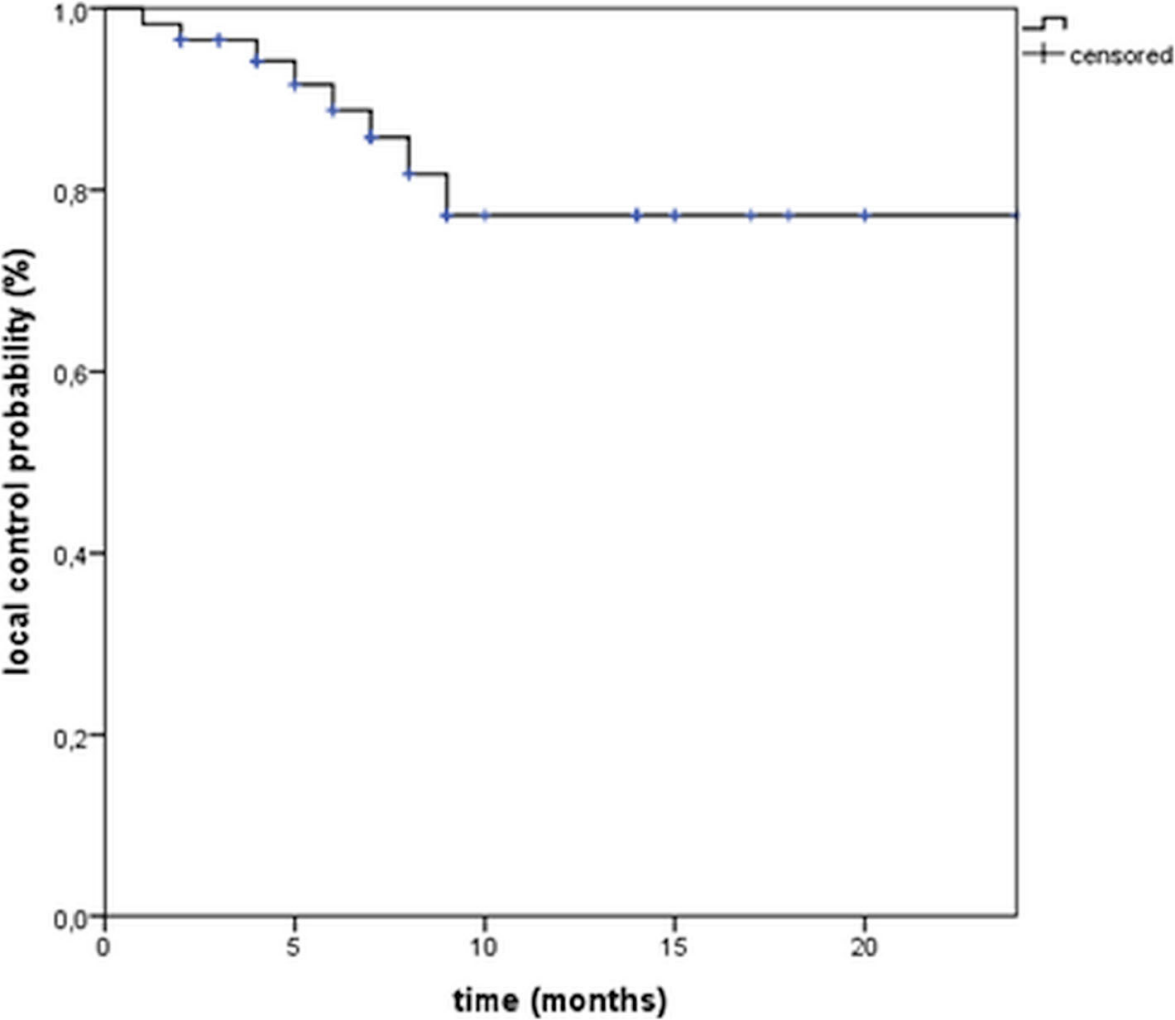
Local control from the time of radiotherapy

**Fig. 2:**
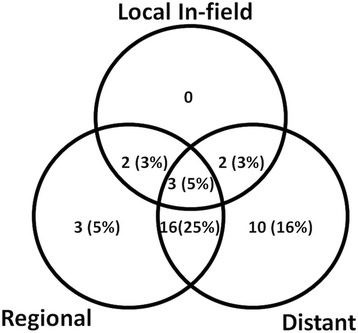
Patterns of failure

Local control at 1 year was 77% from the start of SBRT (Fig. 1). Overall, seven lesions (11%) progressed in field between 3 and 17 months after treatment (median 7 months, 95% CI 5.635–8.365), two in combination with a regional PD (out of field, i.e. liver), two in combination with a distant PD and three of them also in combination with regional and distant metastases. A regional failure in the liver (out of field) was observed in three cases. Sixteen progressed regionally (out of field) in combination with distant metastases and 10 developed only distant metastases (Fig. 2). After PD 11 patients were treated with sorafenib and six patients with TACE and seven patients with regional recurrence were re-irradiated. On univariate analysis the diameter of the tumor and the GTV were associated with improved local control (Table 2). Additionally the EQD2_10_ and consecutively BED_10_ of the prescribed dose, but not the D_max_ or D_95%_, were associated with improved local control (Table 2). The fractionation had no impact on local control and there was no difference concerning LC between patients treated with or without simultaneous integrated protection (SIP, *p* = 0.944, log rank, Table 2). The median progression free survival time was 7 months (95% CI, 5.3–8.6).

**Table 2:** Univariate analysis for local control and overall survival

Variable	Local control			Overall survival		
	HR	95% CI	*p*	HR	95% CI	*p*
CTP	1.725	(0.406–7.335)	0.5	1.806	(0.884–3.691)	0.1
Multifocality	0.696	(0.085–5.688)	0.7	1.471	(0.512–4.228)	0.5
Diameter	1.147	(1.008–1.305)	0.04	1.017	(0.952–1.086)	0.6
Extrahepatic disease	n.a.			0.653	(0.260–1.640)	0.7
Fractionation	0.919	(0.755–1.118)	0.4	0.952	(0.860–1.053)	0.3
SIP	0.950	(0.227–3.989)	0.9	1.298	(0.639–2.637)	0.5
GTV Volume	1.001	(1.000–1.002)	0.008	1.001	(1.000–1.001)	0.008
TVT	0.977	(0.189–5.058)	0.9	1.674	(0.781–3.590)	0.2
EQD2_10, prescribed_	0.952	(0.907–0.998)	0.04	1.002	(0.978–1.026)	0.9
EQD2_10, Dmax_	0.992	(0.967–1.017)	0.5	0.998	(0.988–1.008)	0.7
EQD2_10, D95%_	0.978	(0.919–1.014)	0.4	1.013	(0.990–1.036)	0.3
EQD2_2, mean liver dose_	n.a.			1.009	(0.967–1.054)	0.6
BCLC	n.a.			0.868	(0.428–1.761)	0.7

*CTP* Child-Turcotte-Pugh score, *TVT* Tumor vascular thrombosis, *UVA* univariate analysis, *OS* Overall survival, *LC* Local control, *CI* Confidence interval, *n.a.* not applicable

Dose, Diameter and GTV were considered as continuous variables

### Overall survival

Median overall survival time was 9 months (95% CI, 7.715–10.285). The only significant factor for OS on univariate analysis was the volume of the GTV (Table 2). At the time of the analysis 31 patients (66%) had died, 12 due to disease progression, three of whom due to liver decompensation (3, 6, 10 months after treatment). One patient died due to liver decompensation without PD 4 months after SBRT. Thirteen patients died due other causes (upper gastrointestinal bleed at distance from the PTV *n* = 3, pneumonia *n* = 2, stroke *n* = 1, pulmonary decompensation *n* = 1, urosepsis *n* = 1, cardiac decompensation *n* = 1, sepsis due to liver abscess *n* = 1, renal failure *n* = 3) and five due to unknown causes. The presence of extrahepatic disease had no impact on overall survival (*n* = 9 patients, median OS 9 vs 10 months, *p* = 0.4 log rank, Table 2).

### Toxicity

Three patients with known portal hypertension prior to therapy developed gastric ulcers with bleeding CTC grade 2–3, one, three, four and five months after SBRT which were treated with proton pump inhibitors (2 patients, grade 2) and transfusion (1 patient, grade 3). In the first case the patient who was treated in the past with liver SBRT for another HCC lesion, with an interval of 4 months between the two treatments, developed CTC grade 3 gastroduodenitis requiring a transfusion four months after the second treatment. The Dmax and D 0.5cm^3^ at the stomach at first treatment were 46.6 Gy (EQD2_3_ 64 Gy) and 44.8 Gy (EQD2_3_ 60 Gy) in 12 fractions and in the second treatment 14Gy (EQD2_3_ 12.7 Gy) and 13.4 (EQD2_3_ 12 Gy) in 9 fractions. In this case the addition of the second SBRT contributed probably to the development of the gastrointestinal bleeding. In case of the second patient the Dmax was 33.8 Gy, D0.5cm^3^ 31.7 Gy, D5cm^3^ 28.1 Gy in five fractions (corresponding EQD2_3_ values 66 Gy, 59.2 Gy, 48.4 Gy). All constraints were respected except of the Dmax, which exceeded the constraints by 0.1 Gy. The third patient had a Dmax of 28.8 Gy in 5 fractions, D0.5 cc 25.3 and D5cc 20 Gy (corresponding EQD2_3_ 50.4 Gy, 41 Gy and 28 Gy). The latter had a known gastric antral vascular ectasia (GAVE) which is an uncommon cause of chronic gastrointestinal bleeding. In this case the constraints did not exceed the constraints proposed by Timmerman et al. [13]. An increase of the CTP score was observed in 17 patients of whom 12 patients had PD. Of the five patients without progression 4 had an increase of one point (B7 to B8, A6 to B7, A5 to A6, B8 to B9) and 1 developed an increase of ≥2 points after treatment (A6 to B8) due to a radiation induced liver disease (RILD). The latter recovered fully and died 9 months after SBRT due to renal failure. Only one of these patients, with an increase of one point (A5 to A6) died due to liver decompensation without disease progression 4 months after SBRT. This patient had a 6.6 cm tumor and was treated in 5 fractions with a median dose to the liver of 16 Gy. There were no significant alkaline phosphatase elevations or liver transaminases observed. None of the dose constraints for the liver were violated. One patient developed a necrotic abscess in the PTV of the liver due to a dislocation of a pre-existing stent of the bile duct (Table 3).

**Table 3:** Toxicities, CTCAE >2

A.Toxicitiy	Grade 2	Grade 3	Grade 4	Grade 5
	No (%)	No (%)	No (%)	No (%)
Biochemical				
ALT/AST (u/l)	0	0	0	
Bilirubin (mg/dl)	2 (4%)	6 (13%)	0	
INR	0	0	0	
AP (U/l)	1 (2%)	0	0	
GGT (U/l)	2 (4%)	0	0	
GI-Toxicitiy				
Gastrioduodenitis / GI bleeding	2 (4%)	1 (2%)	0	
Liver-Toxicity				
Abscess			1 (2%)	
RILD		1 (2%)^b^		
Dekompensation				1 (2%)
B.	CTP Deterioration^a^ No (%)			
Score				
1 Point	4			
2 Points	1 (2%)^b^			
Class	2 (4%)			

*GI* gastrointestinal, *CTCAE* Commom Terminology Criteria for Adverse Events (CTCAE) version 4.0, *INR* international normalized ratio, *AST* Alanine aminotransferase, *ALT* Aspartate aminotransferase, *AP* Alkaline phosphatase, *GGT* Gamma-Glutamyltransferase, *RILD* radiation induced liver desease

^a^without progressive disease

^b^same patient

## Discussion

SBRT can lead to excellent results for small HCC tumors (Table 4), but there is little experience with larger tumors. Delivering high doses to large HCCs can be very challenging due to the higher mean liver doses irradiated, that often compromise the liver constraints and thus increase the risk for liver failure, especially in a collective which is highly pre-treated or with a higher Child-Pugh score. As reported by Crane et al. [19] the vast majority of physicians interpret SBRT as meaning doses of radiation (range, 4–20 Gray [Gy]) that may not be ablative but are delivered within about 1 week (i.e., in 3–6 fractions). Adherence to this approach has limited the effectiveness of SBRT for large liver tumors (>7 cm) because of the need to reduce doses to meet organ constraints. Similar to the ongoing LungTech trial delivering eight fractions for central lung tumors (EORTC-22113-08113, EudraCT Number 2012–000415-83) which might be considered as hypofractionation rather than SBRT we therefore chose to use up to 12 fractions. However this allowed the delivering a higher BED_10_ compared to others studies. In a study by Que. et al. [20] reporting results on large HCC (median diameter 11.4 cm) patients were treated with 5 fractions of 5.2–8 Gy resulting to an EQD2_10_ of 33–60 Gy. The local control was modest with 55.5% at 1 year as well as toxicities (1 grade 3 liver enzyme elevation) but only 2 of the 22 patients had a CTP score B. In a prospective Phase I/II study by Bujold et al. tumors with a median size of 7.2 cm were treated with a total dose of 24–54 in 6 fractions with an EQD2_10_ ranging between 28 and 85.5 Gy (median 48 Gy). They reported local control rates of 88% at 1 year. Although all patients had a CTP score A, a CTP score deterioration occurred in 46% of the patients and there were 5 (5%) grade 5 liver failures. In another prospective study from the Princes Margaret Cancer Center, Culleton et al. assessed the outcome of patients treated with SBRT with Child-Pugh B or C HCC (median diameter 5.1 cm), unsuitable for liver transplantation. They treated 29 patients with CTP B HCCs with a median dose of 30 Gy in 6 fractions (median EQD2_10_: 37.5 Gy) and reported high toxicity rates with a decline in CP score by >2 points at 3 months in 63% of the patient. They concluded that SBRT is a treatment option for selected HCC patients with small HCCs and modestly impaired (CP B7) liver function.

**Table 4:** Review of Literature

Author	Study	Nr. of patients	CTP	Diameter (cm)^a^	Fractionation	LC@1 year	mOS	Toxicity
Mendez Romero [23]	Pr.	5 2	A B	4.7	3-5 × 5–12 Gy	75%	22	1 lethal liver failure
Tse [34]	Pr.	31	A	173cm^3b^	6 × 4–9 Gy	65%	11.7	8 grade 3 enzyme elevations, 1 pulmonary embolism, 1 tumor-duodenal connection
Jang [35]	Ret	74 8	A B	3	3 × 11-20Gy	87%@ 2 y.	63%@ 2 y.	5 GI toxicity grade 3 6 CTP elevation >2
Huang [36]	Ret	23 4 1	A B C	4.4	10 × 4.5 Gy 18–20 × 2.5 Gy 18–20 × 1.8 Gy	87.6%	23	1 grade 3 gastric ulcer
Bae [37]	Ret	18 2	A B	<3 cm (80%) 3-5 cm (20%)	5 × 10 Gy	85%	100@ 1 y.	No grade 3 toxicities
Jung [38]	Ret	68 24	A B	8.6 cm^3b^	3-4 × 10-20Gy	92%@ 3 y.		6 patients grade 3 RILD
Wahl [12]	Ret.	57 24 2	A B C	<2 cm (48%) 2-3 cm (26%) 3-5 cm (23%) >5 cm (3.7%)	3-5 × 6–10Gy	97.4%	74%@ 1 y.	1 RILD, 1 GI bleeding 1 worsening ascites
Andolino [9]	Ret.	36 24	A B	3.1 cm	3-5 × 8-16Gy	90%@ 2 y.	48%@ 2 y.	20% CTP progression
Bibault [22]	Ret.	66 9	A B	3.7 cm	3 × 8-15Gy	89.8%	15	5 liver decompensations, 1 grade 4 gastric ulcer, 3 grade 2 duodenal ulcers
Huertas [21]	Ret.	76 11	A B	2.4 cm	3x15Gy	99%	82%@ 1 y.	1 grade 5 hematemesis 2 grade > 3 gastric ulcers
Scorsetti [24]	Ret.	23 20	A B	4.8 cm	3 × 16–25 Gy 6 × 6–10 Gy	86%	18	7 grade > 3 liver enzyme elevations
Seo [39]	Ret.	34 4	A B	40.5ml^b^	3 × 11-12 Gy	79%	32	1 grade 3 soft tissue toxicity
Kwon [40]	Ret.	38 4	A	15.4ml^b^	3 × 10-13Gy	72%	93%@ 1 y.	1 radiation induced hepatic failure
Takeda [41]	Ret.	14 2	A B	1.9-7 cm	5-7 × 5-10Gy	100%	100%	1 RILD
Price [42]	Ret.	14 12	A B	max. 6 cm	3-5 × 8-16Gy	97%	77%	20% CTP worsening
Kang [43]	Pr.	41 6	A B	2.9 cm	3 × 14-20Gy	94%@ 2 y.	68.7@ 2 y.	3 grade 3 GI toxicity, 2 grade 4 gastric ulcers
Su [44]	Ret	114 18	A B	1.1–5.0 cm	1 × 28-30Gy 42–46 Gy in 3–5 fractctions	90%		11 patients hepatic toxicity grade ≥ 3
Kang [45]	Ret	67 34	A B	n.s.	6 fractions	20–29.4 @ 2y	12–15	25 cases deteriorated from grade A to B, 4 from A to C and 6 from B to C
Sanuki [46]	Ret	158 27	A B	2.7 (0.8–5) cm	5 × 7–8 Gy	91% @ 3 y.	70% @ 3 y.	13% acute grade > 3, 2 grade 5 liver failure
Kimura [47]	Ret	56 9	A B	1.6 cm	4 × 12 Gy	100% @ 2 y.	76% @ 2 y.	23% grade > 3
Weiner [26]	Pr.	12	A,B	ca. 5 cm	5 × 8–11 Gy	91%	38%@ 1 y.	9 CTP decline 2 grade 5hepatic failure
Que [48]	Ret	104 11	A B	≤ 4 cm (35%) 4–9 cm (41%) ≥10 cm (24%)	26–40 Gy in 3–5 fract.	85%	15	3 patients grade 5 25 patients grade 3
Que [27]	Ret.	22 2	A B	11.4 cm	5 × 5.2-8Gy	55.6%	11	1 grade 3 liver enzyme elevation
Bujold [10]	Pr.	102 0	A B	7.2 cm	6 × 4-9Gy	88%	17	6 grade > 3 liver failures, 1 grade 5 cholangitis, 1 grade 5 GI bleed 16 grade ≥ 3 enzyme elevations
Culleton [33]	Pr./ Ret.	0 29	A B	5.1 cm	5–15 fractions 19.7–46.8Gy^c^	n.a.	7.9	63% CTP decline ≥ 2 points, 5 grade 3 thrombocytopenia, 3 > grade 3 elevation of liver enzymes
Current study	Ret	28 19	A B	7 cm	3-12 × 4-15Gy	77%	9	1 RILD, 1 grade 5 liver decompensation, 1 grade 3 GI-bleed, 1 necrotic abscess

^a^median

^b^Volume

^c^Min dose to 95% to PTV

*CTP score* Child-Turcotte-Pugh score, *LC @ 1 year* local control at 1 year, *mOS* median overall survival, *GI* Gastro-intestinal, *RILD* Radiation induced liver disease

Compared with the study of Bujold et al. we could show favorable local control rates of 77% at one year (1-y-LCR), Table 4, [9], [12], [21–25] with a lower toxicity profile although the tumors were of similar size, the EQD2_10_ delivered was higher, the Child-Pugh score was higher and the patients were heavily pre-treated including re-SBRT and SIRT. This is probably due to the use of a more moderate fractionation, without compromising the dose constraints for the OARs and in cases were the constraint could not be met, the use of SIP, that allowed the delivery of a lower dose to small subvolumes without compromising the dose to the hole PTV, while staying within tolerance of the OARs. Neither the use of SIP nor the use of 12 fractions had a negative impact on local control or overall survival. In this way we achieved a higher median EQD2_10_: 63 Gy when compared with other groups treating large HCCs with more moderate toxicities. Similar to our approach, Crane et al. [19], suggested a simultaneous integrated boost (SIB) with simultaneous integrated protection (SIP) for large liver tumors treated with SBRT.

The overall survival in our analysis was intermediate due to several factors such as multiple pre-treatments (resection, TACE, sorafenib, SBRT, SIRT), the presence of a CTP score B in 40% at the time of treatment, the presence of tumor vascular thrombosis in 28% and the presence of extrahepatic disease in 19%.

In a propensity score matching comparing long term survival between patients treated either with transarterial or surgical resection for huge hepatocellular carcinoma [28] the one year survival rate in the surgery group was 69.7% and in the TACE group 40.2% which is similar to our data. For patients with oligometastatic disease (BCLC stage C) there is emerging evidence that TACE, a local ablative treatment, significantly improves OS [17, 18], Bettinger submitted). Taking these data into consideration, patients that progressed under TACE were also offered SBRT by the MDT panel, as an alternative to systemic treatment. Furthermore, according to Crane et al. [19], the rationale for taking an aggressive approach to treating large liver tumors is that patients often die from liver failure related to disease progression regardless of the presence of extrahepatic disease. As all recurrences observed in our analysis occurred in combination with either regional progression in the liver or distant progression, combining Sorafenib with SBRT, aiming to enhance the efficacy of SBRT while treating extrahepatic disease, seemed promising. This hypothesis was investigated in a phase I trial by the group of the Princess Margaret Cancer Center, [29, 30]. The concurrent use of sorafenib with SBRT resulted in unacceptably high rates of serious toxicity and is not recommended for further testing, particularly in patients in whom irradiation of a large amount of liver. Currently a randomized phase III trial (RTOG 1112) is testing the role of SBRT followed by sorafenib vs sorafenib alone in terms of overall survival improvement.

A number of limitations applying to our study need to be considered, including the retrospective and single-institution nature of our study and the small sample size tested. As such, these results should be interpreted with caution and only hypothesis generating.

In the pre SBRT era radiotherapy was considered a treatment with high toxicity rates due to the underlying liver disease in most of the patients. Today, there is growing evidence [10, 31] that SBRT for hepatocellular carcinoma is a safe treatment option with toxicity rates ranging from 0 to 36% [9, 10] and radiation induced liver disease is less common <5% in experienced hands.

Furthermore radiotherapy is a very well tolerated treatment in terms of quality of life [31, 32] with the only observed deficits being temporary worsening of appetite and fatigue. In the prospective study of Klein et al. [31] overall quality of life did not decline and baseline overall QOL predicted improved survival. Stereotactic body radiation therapy is well tolerated and warrants comparison against other liver-directed therapies.

## Conclusion

In conclusion, our results are highly concordant with published literature regarding local control for smaller tumors. We could show a good overall toxicity profile with only a slightly shorter overall survival in patients with high CTP score which is an unfavorable prognostic factor [33], pre-treatment, multifocality, frequent TVT and tumor volume. Furthermore we have also included patients which were re-irradiated and tolerated SBRT without major complications. SBRT is a feasible treatment for and warrants greater recognition as a treatment option in the management of this malignancy. In this context we are now conducting, a phase II single institutional prospective comparison between TACE and SBRT (HERACLES, DRKS number: DRKS00008566).

## Additional file

Additional file 1: Supplementary table showing the fractionation regimes used. (DOCX 13 kb)

## Abbreviations

4D CT: 4 dimensional-CT
BCLC: Barcelona-Clinic Liver Cancer
BED: Biological effective doses
CBCT: Cone beam computed tomography
CTP: Child-Turcotte-Pugh
ECOG: Eastern cooperative oncology group
EQD2: Equieffective doses for 2 Gy fractions
GTV: Gross tumor volume
HCC: Hepatocellular carcinoma
IMRT: Intensity modulated radiotherapy
ITV: Internal target volume
LC: Local control
MDT: Multidisciplinary board
OARs: Organs at risk
RECIST: Response evaluation criteria in solid tumors
RFA: Radiofrequency ablation
RILD: Radiation induced liver disease
SBRT: Stereotactic body radiotherapy
SIP: Simultaneous integrated protection
SIRT: Selective internal radiation therapy
SOPs: Standard operating procedures
TACE: Transarterial chemoembolization
