# Supplementary material for: Excellent local control and tolerance profile after stereotactic body radiotherapy of advanced hepatocellular carcinoma
Source: Radiat Oncol. 2017 Jul 12;12:116. doi: 10.1186/s13014-017-0851-7 (PMC5508695; doi:10.1186/s13014-017-0851-7)
Supplement: Additional file 1: — Supplementary table showing the fractionation regimes used. (DOCX 13 kb) [file 13014_2017_851_MOESM1_ESM.docx]

Additional file

Supplementary table showing the fractionation regimes used.

|  | **All lesions** | **CTP A** | **CTP B** |
| --- | --- | --- | --- |
| **Variable** | **No.** | **No.** | **No.** |
| **Fractionation ****  **10-12 * 4-5.5 Gy**  **5 * 7-11 Gy**  **3 * 12.5-15 Gy** | 24  31  9 | 16  18  7 | 8  13  2 |

** Per lesion

CTP: Child-Turcotte-Pugh score
